# Monitoring of Waterborne Parasites in Two Drinking Water Treatment Plants: A Study in Sarawak, Malaysia

**DOI:** 10.3390/ijerph13070641

**Published:** 2016-06-28

**Authors:** Reena Leeba Richard, Init Ithoi, Mohamad Azlan Abd Majid, Wan Yusoff Wan Sulaiman, Tian Chye Tan, Veeranoot Nissapatorn, Yvonne Ai Lian Lim

**Affiliations:** Department of Parasitology, Faculty of Medicine, University of Malaya, Kuala Lumpur 50603, Malaysia; reenarichard88@gmail.com (R.L.R.); init@um.edu.my (I.I.); azlan.jaime@gmail.com (M.A.A.M.); wanyus@um.edu.my (W.Y.W.S.); tantianchye@um.edu.my (T.C.T.); veeranoot@um.edu.my (V.N.)

**Keywords:** waterborne parasites, water parameters, drinking water treatment plants, processing sites, Malaysia

## Abstract

The occurrence of waterborne parasites coupled with water parameters at various processing sites of two drinking water treatment plants (A and B) and seven distribution system (DS) sites in Sarawak, Malaysia were studied. Ten liters of water underwent immunomagnetic separation (IMS) technique to detect the presence of *Giardia* and *Cryptosporidium* (oo)cysts. The remaining supernatant was used to detect other parasites whilst 50 mL of water sample was each used in the detection of free-living amoebae and fecal coliforms. Sampled water was positive for *Giardia* (32.9%; 28/85), *Cryptosporidium* (18.8%; 16/85) followed by *Spirometra* ova-like (25.9%; 22/85), *Blastocystis*-like (25.9%; 22/85), nematode larvae-like (8.2%; 7/85) and *Taenia* ova-like (1.2%; 1/85). Meanwhile, 90.2% (55/61) samples were positive for *Acanthamoeba* and *Naegleria* via cultivation and of these, 11 isolates were confirmed as *Acanthamoeba* genotype T3 (5/7) and T4 (2/7) followed by *Naegleria* sp. (4/11), *Naegleria italica* (2/11), *Naegleria australiensis* (1/11), *Naegleria angularis* (1/11) and *Vahlkampfia* sp. (3/11). *Cryptosporidium*, *Acanthamoeba* and *Naegleria* were also detected in one of the seven tested DS sites. Only *Giardia* and *Cryptosporidium* showed significant correlations with fluoride and fecal coliforms. These results describe the occurrence of waterborne parasites that will assist key stakeholders in mitigating contamination at the specific sites.

## 1. Introduction

Safe potable drinking water is vital to human life and its quality is of great public concern, not only to the consumers, but also to water suppliers and authorities. World Health Organization/United Nations Children’s Fund (WHO/UNICEF) [1] stated that an estimated 1.1 billion people do not have access to improved water supplies [2]. Moreover, Clasen and Bastable [3] reported hundreds of million people consumed water that was contaminated during collection, transport and storage. Surface water (i.e., rivers, lakes, reservoirs, and basins) and groundwater are the two main sources of water supplies. Numerous studies have been conducted globally to investigate the quality of untreated (lake water) and treated (municipal water) water supplied to the consumers. The supply of safe drinking water is crucial and it requires multiple barriers to prevent the entry and transmission of pathogens. Water supplies are routinely checked to ensure that water is safe for consumption via water treatment processes that include a series of treatment processes (e.g., coagulation, flocculation, clarification through sedimentation, filtration and disinfection) that help in the reduction of microorganisms that pose threat to public health [4]. 

The increasing world population coupled with industrialization and urbanization will have serious impact on the availability of safe drinking water supply, hence, contributing to a rapid growth in waterborne disease outbreaks [5,6,7]. The possibility of contaminated drinking water affecting large number of people has been reported and has caused various health hazards, and may lead to major illnesses. A review done by Karanis et al. [8] reported that *Giardia duodenalis* and *Cryptosporidium parvum* accounted for a majority of outbreaks worldwide with 40.6% and 50.8%, respectively, followed by other protozoan parasites such as *Entamoeba*, *Cyclospora*, *Toxoplasma*, *Isospora*, *Blastocystis* and *Balantidium*. Both *Giardia* and *Cryptosporidium* are parasitic intestinal protozoa, transmitted via fecal–oral route, which cause giardiasis and cryptosporidiosis, respectively. They remain public health concerns, as demonstrated by continued outbreaks [9,10,11,12,13,14,15]. Webb [16] reported that four members of a group of 22 travellers from Kansas, USA were confirmed with giardiasis and two probable cases were identified while travelling to a resort in Mexico whilst a recent outbreak caused by cryptosporidiosis occurred in Germany following an extreme river flooding with 24 cases being notified [17].

In addition, several studies have also documented outbreaks caused by free-living amoebae (FLA) such as *Acanthamoeba* and *Naegleria* that are mostly found in the environment [18] and can be isolated from soil, air, water, dust, sewage and sediments [19]. The genera *Acanthamoeba* causes amoebic keratitis (AK) linked to the usage of non-sterile saline solutions for contact lenses with a recent outbreak reported in Singapore and America [20,21] as well as central nervous system lethal infections such as granulomatous amoebic encephalitis (GAE) [22]. Meanwhile, the only *Naegleria* species that is pathogenic to humans, *Naegleria fowleri*, is able to cause primary amoebic meningoencephalitis (PAM). During 2008 to 2009, there were 13 positive cases of *Naegleria fowleri* infections in Karachi, Pakistan [23]. The patients were believed to be in contact with contaminated pipe water during ritual ablution. 

There have been numerous studies conducted throughout the world on waterborne parasites and FLA taken from environmental samples such as rivers, lakes and other recreational areas including samples from wastewater. However, limited studies are available in drinking water treatment plants. In Malaysia, previous studies documenting on the occurrence of protozoan parasites, specifically, *Giardia* and *Cryptosporidium* (oo)cysts, in drinking water treatment plants were reported by Ahmad et al. [24] and Tan [25]. Meanwhile, no studies have been carried out on free-living amoebae. Furthermore, there is a scarcity of information regarding drinking water quality in East Malaysia, especially in Sarawak and Sabah. The aim of this study is to determine the occurrence of waterborne parasites (*Giardia* and *Cryptosporidium*) and free-living amoebae (*Acanthamoeba* and *Naegleria*) in drinking water at various processing sites of two major drinking water treatment plants in Sarawak. Qualitative data on the detection of fecal coliform and other parasites were also collected to improve understanding on the types of microbial contamination in these treatment plants. 

## 2. Experimental Section

### 2.1. Study Background, Design and Sampling Sites

Prior to the commencement of this study, ethical consideration (MEC ref. no. 908.2) was obtained from the Ethics Committee of the University Malaya Medical Centre (UMMC) Malaysia. Permission to conduct this study was also obtained from the relevant local water authorities prior to sample collection. 

Sarawak, the largest state in Malaysia, with a total area of 124,449 km^2^ [26], is home to an overall population of 2,471,140 people [27] with sporadic occurrence of dry and wet season. Kuching, the state capital, with approximately 705,546 people [27], is located on the western coast of Sarawak. Since its independence in 1963, Kuching has since developed into a modern city in terms of economic growth. Due to rapid growth of urbanization and infrastructure, environmental changes such as erosion and water pollution have gradually increased leading to negative effects towards gaining clean, potable water. In Sarawak, rivers are still considered the main raw water sources, which still depend heavily on rainfall with an annual mean of 3861 mm [26]. Hence, contamination, which occurs in surface water, especially river, requires appropriate treatment to control potential agents that cause diseases. 

**Figure 1:**
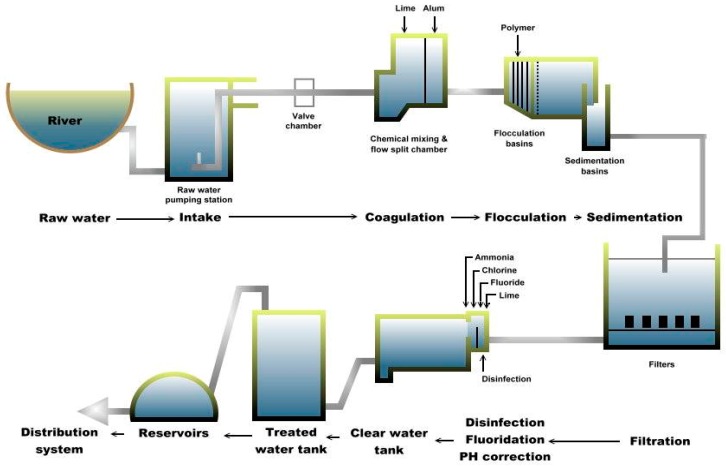
Schematic diagram of Plant A.

Water samples were collected on separate occasions at each of the two drinking water treatment plants (A and B) in Sarawak. Plant A is divided into two subdivisions (A-1 and A-2) and each subdivision is further divided into six processing sites, namely raw, coagulation, flocculation, sedimentation, filtration and treated water sites, as shown in Figure 1. These subdivisions are part of the plant’s design and its raw water is from the same source. Plant A is situated near the bank of the Sungai Sarawak Kiri branch of the Sarawak River (total length of 120 km). Currently, raw water from plant A is pumped from Sungai Sarawak Kiri to the treatment plant from four intakes and further undergoes conventional treatment processes before being supplied to the consumers [28]. Thus, plant A consists of comprehensive treatment processes to meet the increasing water demands. As of 2014, plant A supplied more than 99.07% of the total water production for the main city with a total capacity of more than 500 megaliters per day (MLD). 

**Figure 2:**
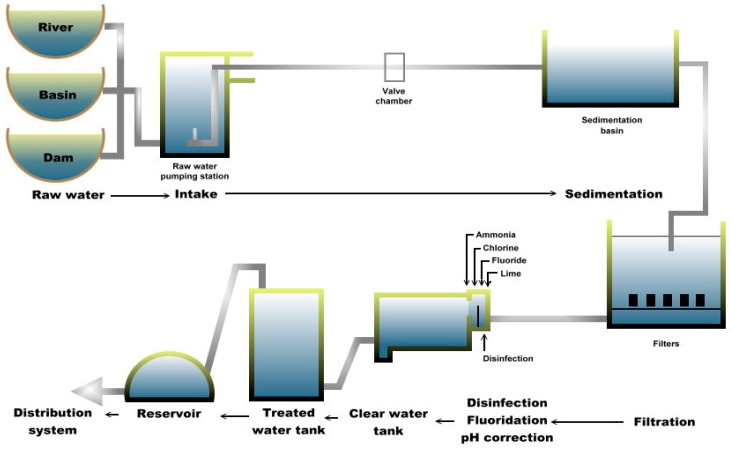
Schematic diagram of Plant B.

Meanwhile, plant B consists of three different types of raw water, namely from river (raw-1), basin (raw-2) and dam (raw-3). These raw water sources are accumulated and transferred into each process that includes sedimentation, filtration and treatment (Figure 2). Plant B, located on a hill (approximately 20 km from plant A), was the original waterworks constructed during Brooke’s administration (in the late 19th century) and chlorination was introduced in 1960. The river originates from a nearby mountain and flows past villages. Previously, the plant depended only on rainfall; hence, the development of the dam and the basin catchment (both built in the 1970s) helped in improving the reliability of water supply. Finished water from plant B accounted for about 0.93% of the total water production as of 2014 [29] with a total capacity of 16 MLD that flows by gravity into the distribution system. Although plant B has only basic treatment processes and is smaller than plant A, plant B continues to be an important supplier of treated water to certain areas of the main city.

Multiple-barrier approach is necessary to minimize potential pathogens. Once the raw water enters the treatment plant, it undergoes coagulation to begin removal of dirt and other particles by adding alum, polymer and lime to form “floc”. The small suspended particles will clump together and due to the combined weight, the particles will settle at the bottom as sediment. This process is referred to as the flocculation process. Then, in the sedimentation process, the “floc” settles to the bottom. The clarified water then flows to the filtration process section and is passed through sand filters to assist in removing the remaining particles. This process enhances the effectiveness of the disinfection process. Subsequently, chlorine is added not only to the water supply in the plant but also in the distribution pipes to control remaining microorganisms. Chlorine is only partially effective against *Giardia* and ineffective against *Cryptosporidium*. Fluoride is also added to prevent tooth decay and act as a rust inhibitor to preserve the pipes that deliver potable water. After various treatment processes, the treated water is then transferred and accumulated at the distribution system (DS) sites and piped out to the consumers. In addition, more than 100 distribution system sites are located within the city but only seven sites were selected for this study based on logistical reasons and permission obtained.

### 2.2. Measurement of Water (Physical and Chemical) Parameters

The physical and chemical parameters were measured in situ at each of the processing site prior to the collection of water samples. The physical parameters such as temperature, pH, conductivity, total dissolved solids (TDS), salinity (SAL), dissolved oxygen (DO) and oxidation reduction potential (ORP) were measured using multi-probe parameter (YSI 556 Multiprobe System, Yellow Springs, OH, USA). Meanwhile, turbidity was measured using turbidity meter (Hach 2100P Portable Turbidimeter, Loveland, CO, USA) and chemical parameters such as ammonia, chlorine, fluoride, nitrate and nitrite were measured using colorimeter (Hach DR/890 Portable Colorimeter, Loveland, CO, USA) in the laboratory. The parameters were then recorded as mean values at each sampling site.

### 2.3. Collection and Filtration of Water Samples

This study was carried out on separate occasions during a course of 15 months from July 2012 to October 2013. A total of 10 L of water samples were collected in sterile polyethylene containers from each sampling site of the water treatment plants and these samples were then filtered at the laboratory. For (oo)cysts detection, each sample was filtered separately through a nitrocellulose membrane (1.2 µm pore size, 142 mm diameter, Millipore, MA, USA). The deposited materials were detached and washed from the membrane filter using 10% Tween 80 and decanted into a 50 mL centrifuge tube. In addition, two 50 mL water samples were taken from each site for the detection of fecal coliform and free-living amoebae. The samples for the detection of *Giardia* and *Cryptosporidium* (oo)cysts as well as fecal coliform were packed in cold polystyrene box (12 ± 2 °C) while samples for FLA detection were kept at room temperature. All samples were transferred by air and further processing took place at the Department of Parasitology, Faculty of Medicine, University of Malaya, Kuala Lumpur, Malaysia at the soonest time possible after collection.

### 2.4. Detection of Giardia and Cryptosporidium (Oo)cysts

After washing the membrane filter, the sample was concentrated by centrifugation at 3500 rpm for 10 min (Kubota Corporation, Tokyo, Japan). The top supernatant was discarded before being brought down to 10 mL. The eluate was then purified using immunomagnetic separation (IMS) technique (Dynabeads GC-Combo, Invitrogen, Carlsbad, CA, USA) applied according to USEPA Method 1623.1 [30] with minor modifications. The iron beads coated with *Giardia*- and *Cryptosporidium*-specific monoclonal antibodies were used to separate the (oo)cysts from water sediment and debris using the magnetic particle concentrator. The remaining supernatant was then used to further detect other parasites. Final volume of 50 µL sample was deposited onto a microscope well slide (Dynabeads Spot-On, 9 mm single well, Life Technologies, Carlsbad, CA, USA). 

The slide was then air-dried and fixed with methanol. Sample was stained with commercial fluorescence isothiocyanate (FITC)-labelled (*Crypto*/*Giardia* Cel IF Kit, Cellabs Pty Ltd., Brookvale, Australia) according to the manufacturer’s instructions. The sample was further stained with 4′6-diamidino-2-phenyl indole (DAPI) (Sigma Chemicals, Perth, WA, Australia) and washed with phosphate buffer saline (PBS) and finally with distilled water. Mounting medium was then added. Nail polish was used to seal the coverslip and left to dry. Observation was done under 400× magnification via epifluorescence microscope (Olympus BX51, Tokyo, Japan) to confirm the presence of (oo)cysts. Round, oval or ellipse shapes with bright apple-green fluorescence were identified (*Giardia* cyst: 8 to 18 µm × 5 to 15 µm; *Cryptosporidium* oocyst: 4 to 6 µm). The slide that was found to be positive via FITC (fluorescein isothiocyanate) was then examined through DAPI (4’, 6-diamidino-2-phenylindole) filter to detect the presence of sky blue nuclei. Positive and negative staining controls were also included in each analysis. (Oo)cysts on the slides were identified and counted at least two times to minimize error (calculation, e.g., 1 cyst of *Giardia* found in a volume of 50 µL (which represents 10 L of samples) equals to 0.1 cyst/L).

### 2.5. Detection of Other Parasites (from IMS Supernatant)

The remaining supernatant from IMS steps was poured into a 15 mL centrifuge tube. The decanted sample was centrifuged at 2500 rpm for 10 min to detect the presence of other parasites. Approximately 50 µL of sediment was placed on a glass slide and stained with one drop of Lugol’s iodine solution. After the slide was covered with a coverslip, it was observed under a light microscope under 100× and 400× magnification. The observation of all putative parasites was recorded.

### 2.6. Cultivation of Acanthamoeba and Naegleria on Solid Agar 

Fifty (50) mL sample was concentrated by centrifugation at 2500 rpm for 15 min (Kubota Corporation, Tokyo, Japan) before being brought down to 5 mL and re-suspended several times. A volume of 1 mL was added onto the surface of non-nutrient agar (NNA)-*Escherichia coli* (*E. coli*) plates. The preparation of NNA-*E. coli* was carried out as reported by Init et al. [31]. The duplicate plates of each sample were incubated at room temperature (25 ± 3 °C) and examined daily up to 14 days using an inverted microscope (Nikon, Tokyo, Japan). Once growth was spotted, a small piece of the agar containing the amoebae was excised and cultured onto a fresh, new NNA-*E. coli* plate. Sub-culturing of each sample was done several times until a monogenous culture was obtained. 

### 2.7. Detection of Acanthamoeba and Naegleria Species by Molecular Analysis 

Eleven isolates of *Acanthamoeba* and *Naegleria*, respectively, obtained from selected sites of plant A (1 pooled sample of raw water and 4 treated water samples), plant B (3 samples of raw water and 2 treated water samples) and distribution system (1 sample) underwent further molecular identification and subjected to deoxyribonucleic acid (DNA) extraction using QIAamp DNA blood mini kit (Qiagen, Hilden, Germany) prior to polymerase chain reaction (PCR) for the detection of *Acanthamoeba* and *Naegleria* species. The confirmation of *Acanthamoeba* species and genotype were performed using *Acanthamoeba* specific primer pair [32] which included the forward primer (JDP1), 5′-GGCCCAGATCGTTTACCGTGAA-3′ and the reverse primer (JDP2), 5′-TCTCACAAGCTGCTAGGGAGTCA-3′. Amplification reactions were performed in a volume of 30 µL which contained distilled water, 10× DNA polymerase buffer (Thermo Scientific, Vilnius, Lithuania), 25 mM MgCl_2_ (Thermo Scientific, Vilnius, Lithuania), 10 mM deoxynucleotide triphosphate (dNTP) mix (Thermo Scientific, Vilnius, Lithuania), forward and reverse primer, 1 U/µL Taq DNA polymerase (Thermo Scientific, Vilnius, Lithuania) and DNA template. The reaction for the primer set involved a pre-PCR heat cycle at 94 °C for 5 min, followed by 40 cycles at 94 °C for 1 min, 60 °C for 1 min, 72 °C for 1 min and an extension at 72 °C for 5 min. 

Meanwhile, two sets of primer pairs designed from ITS regions were used, and *Naegleria* genus-specific (5′-GAACCTGCGTAGGGATCATTT-3′ and 5′-TTTCTTTTCCTCCCCTTATTA-3′) and *Naegleria fowleri* species-specific (5′-GTGAAAACCTTTTTTCCATTTACA-3′ and 5′-AAATAAAAGATTGACCATTTGAAA-3′) [33]. Amplification reactions were performed in a volume of 30 µL which contained distilled water (dH_2_O), 10× DNA polymerase buffer (Thermo Scientific, Vilnius, Lithuania), 25 mM MgCl_2_ (Thermo Scientific, Vilnius, Lithuania), 10 mM deoxynucleotide triphosphate (dNTP) mix (Thermo Scientific, Vilnius, Lithuania), forward and reverse primer, 1 U/µL Taq DNA polymerase (Thermo Scientific, Vilnius, Lithuania) and DNA template. The reaction for both primer sets involved a pre-PCR heat cycle at 94 °C for 5 min, followed by 35 cycles at 95 °C for 30 s, 55 °C for 30 s, 72 °C for 45 s and an extension at 72 °C for 5 min. 

For negative control, the DNA template was replaced with the same volume of distilled water. PCR amplicon was separated by a 1.5% agarose gel prepared in 1X of tris-acetate-EDTA (TAE) as running buffer and the electrophoresis was run at 100 V for 45 min. GeneRuler 100 bp DNA ladder Plus (Thermo Scientific, Vilnius, Lithuania) was used to compare the amplicon sizes. Post-staining method was then carried out using ethidium bromide and gel was viewed under UV-transilluminator. Positive sample was then send for sequencing and homology search was performed using Basic Local Alignment Search Tool (BLAST) software from National Center for Biotechnology Information (NCBI) homepage as mentioned by Altschul et al. [34]. The alignments were obtained with ClustalW and edited with BioEdit. Once the consensus sequences were blasted and retrieved from GenBank database, phylogenetic analysis were performed using neighbour-joining and maximum-likelihood of MEGA version 6 software, followed by Kimura 2-parameter algorithm with bootstrap analysis of 1000 replicates [35]. The reference isolates of *Acanthamoeba castellanii* genotype T1 (GenBank:U07400), *Acanthamoeba palestinensis* genotype T2 (U07411), *Acanthamoeba griffini* genotype T3 (U07412), *Acanthamoeba triangularis* genotype T4 (AF346662), *Acanthamoeba lenticulata* genotype T5 (U94739), *Naegleria italica* (GU597047), *Naegleria gruberi* (AJ132022), *Naegleria schusteri* (AJ566626), *Naegleria philippinensis* (AY033618), *Naegleria australiensis* (AB128053), *Naegleria fowleri* (AJ132027), *Naegleria lovaniensis* (X96569), *Naegleria pussardi* (X96571), *Naegleria angularis* (AJ785756), *Allovahlkampfia spelaea* (EU696949), *Vahlkampfia avara* (AJ698837) and *Vahlkampfia orchilla* (AJ973127) were used. *Balamuthia mandrillaris* (AF477022) and *Hartmannella vermiformis* (AF426157) were selected as the outgroup.

### 2.8. Isolation of Fecal Coliform

Each sample was filtered using a vacuum pump through a sterile nitrocellulose membrane filters (0.45 µm pore size, 47 mm diameter, Millipore, Bedford, MA, USA) which was held in a filter holder. The membrane was transferred to a sterile petri dish with absorbent pad (Millipore, Bedford, MA, USA) containing lauryl sulfate membrane medium (Oxoid, Hampshire, UK) agar plates. The plate was sealed with parafilm and incubated at 30 °C for 4 h to resuscitate the growth of bacteria before further incubation at 44.5 °C for 14 h. Fecal coliform that formed yellow colonies were counted and expressed as colony forming unit (CFU) per 100 mL (CFU/100 mL).

### 2.9. Statistical Analysis

Data obtained were analyzed using Statistical Package for Social Sciences version 16.0 for Windows (SPSS Inc., Chicago, IL, USA) software. The association between the occurrence of waterborne parasites and water parameters were determined by using bivariate correlation and linear regression analysis. The values of *p* < 0.01 or *p* < 0.05 were considered as statistically significant. 

## 3. Results

### 3.1. Overall Occurrence of Parasites Detected via Microscopy at Each Drinking Water Treatment Plant and the Distribution Systems

The occurrence of parasites detected from various processing sites of the two major drinking water treatment plants (A and B) and distribution system (DS) sites in Sarawak, Malaysia is shown in Table 1. *Giardia* cysts (32.9%; 28/85) were found in the highest percentage of occurrence in comparison to other parasites (31.8%; 27/85) followed by *Cryptosporidium* oocysts (18.8%; 16/85). Meanwhile, amoebae (*Acanthamoeba* and *Naegleria*) were detected in 90.2% (55/61) of samples. 

### 3.2. Occurrence and Concentration of Giardia Cysts and Cryptosporidium Oocysts at Each Treatment Plant

The occurrence of *Giardia* cysts and *Cryptosporidium* oocysts in each treatment plant is shown in Table 1. In subdivision A-1, *Giardia* cysts were found positive in 20.0% (6/30) of the samples compared to *Cryptosporidium* oocysts with 16.7% (5/30) while in subdivision A-2, *Giardia* cysts were detected in 43.3% (13/30) of the samples in comparison to *Cryptosporidium* oocysts with 20.0% (6/30). Similarly in plant B, *Giardia* cysts (50.0%; 9/18) were also found higher in comparison to *Cryptosporidium* oocysts that accounted for 22.2% (4/18) of the samples. With regards to the distribution in each processing sites, *Giardia* cysts and *Cryptosporidium* oocysts were detected in most of the sites, as shown in Table 2. The (oo)cysts were found sporadically in filtration and treated water sites. 

In terms of (oo)cysts concentrations, *Giardia* cysts (0.18 ± 0.35 cysts/L) were found highest in raw water samples obtained from subdivision A-1 compared to *Cryptosporidium* oocysts (0.02 ± 0.04 oocysts/L). Subsequently, in coagulation site, *Giardia* cysts (0.06 ± 0.05 cysts/L) were also detected higher in comparison to *Cryptosporidium* oocysts (0.04 ± 0.09 oocysts/L). Both *Giardia* (0.02 ± 0.04 cysts/L) and *Cryptosporidium* (0.02 ± 0.04 oocysts/L) were equally detected in flocculation site. Water samples from sedimentation site showed positive for *Cryptosporidium* oocysts (0.06 ± 0.09 oocysts/L) but none were found in samples from filtration and treated water sites. In addition, *Giardia* cysts were not detected in sedimentation, filtration and treated water sites. *Giardia* cysts revealed the highest overall mean ± SD with 0.04 ± 0.15 cysts/L (range of concentration: 0 to 0.80 cysts/L) in comparison to *Cryptosporidium* oocysts with (0.02 ± 0.06 oocysts/L; range of concentration: 0 to 0.20 oocysts/L).

Meanwhile, for raw water in subdivision A-2, *Giardia* cysts (0.44 ± 0.53 cysts/L) were found highest in comparison to *Cryptosporidium* oocysts (0.04 ± 0.05 oocysts/L). Both *Giardia* cysts (0.14 ± 0.11 cysts/L) and *Cryptosporidium* oocysts (0.14 ± 0.19 oocysts/L) were detected in coagulation site. Subsequently, *Giardia* cysts (0.18 ± 0.20 cysts/L) were also detected higher in flocculation site compared to *Cryptosporidium* oocysts (0.04 ± 0.05 oocysts/L). *Giardia* cysts (0.06 ± 0.13 cysts/L) were found in sedimentation site but no oocysts were detected throughout sedimentation, filtration and treated water processing sites. In addition, *Giardia* cysts were not present in filtration process but were detected in treated water (0.02 ± 0.04 cysts/L). *Giardia* cysts revealed the highest overall mean ± SD with 0.14 ± 0.27 cysts/L (range of concentration: 0 to 1.20 cysts/L) in comparison to *Cryptosporidium* oocysts (0.04 ± 0.09 oocysts/L; range of concentration: 0 to 0.40 oocysts/L).

In plant B, raw-1 site was contaminated with *Giardia* cysts (0.17 ± 0.29 cysts/L) and *Cryptosporidium* oocysts (0.03 ± 0.06 oocysts/L) while raw-2 site was found positive for *Giardia* cysts (0.17 ± 0.12 cysts/L) but no oocyst was detected. Meanwhile, for raw-3 site, *Giardia* cysts (0.17 ± 0.21 cysts/L) were detected higher in comparison to *Cryptosporidium* oocysts (0.07 ± 0.06 oocysts/L). Both *Giardia* cysts (0.03 ± 0.06 cysts/L) and *Cryptosporidium* oocysts (0.03 ± 0.06 oocysts/L) were equally detected in sedimentation process. *Giardia* cysts were found in filtration (0.03 ± 0.06 cysts/L) and treated water sites (0.13 ± 0.23 cysts/L) but no oocysts were detected in both processing sites. Similar to subdivisions A-1 and A-2, *Giardia* cysts revealed the highest overall mean ± SD in plant B with 0.12 ± 0.17 cysts/L (range of concentration: 0 to 0.50 cysts/L) compared to *Cryptosporidium* oocysts (0.02 ± 0.04 oocysts/L; range of concentration: 0 to 0.10 oocysts/L).

### 3.3. Detection of Other Parasites at Each Treatment Plant

Several parasites were detected from the remaining IMS supernatant and results are as shown in Table 1. These parasites were detected highest in subdivision A-1 (40.0%; 12/30) followed by subdivision A-2 (30.0%; 9/30) while in plant B, only 3.3% (6/18) showed positive for other parasites.

In subdivision A-1, *Blastocystis*-like and nematode larvae-like were detected in raw water while *Blastocystis*-like and *Spirometra* ova-like were found positive in coagulation site. In addition, *Spirometra*-ova like and nematode larvae-like were also found in water samples from flocculation site. *Blastocystis*-like were found in both sedimentation and filtration processing sites. 

Meanwhile, in subdivision A-2, *Blastocystis*-like, *Spirometra* ova-like and nematode larvae-like were found in raw water but only *Spirometra* ova-like was found in coagulation site. *Spirometra* ova-like and nematode larvae-like were detected from water samples obtained from flocculation site while *Blastocystis*-like and *Spirometra* ova-like were found positive in sedimentation site. Nematode larvae-like was only found positive in filtration site. 

For plant B, raw-1, raw-2 and raw-3 sites showed positive for *Blastocystis*-like and *Spirometra* ova-like. Subsequently, *Spirometra* ova-like was only found positive in sedimentation site while *Taenia* ova-like and *Spirometra* ova-like were detected in filtration processing site. Overall, *Spirometra* ova-like (25.9%; 22/85), *Blastocystis*-like (25.9%; 22/85), nematode larvae-like (8.2%; 7/85) and *Taenia* ova-like (1.2%; 1/85) were detected in various sites of the plants except for treated water. 

### 3.4. Microscopic Observation and Molecular Analysis of Amoebae Isolates

**Figure 3:**
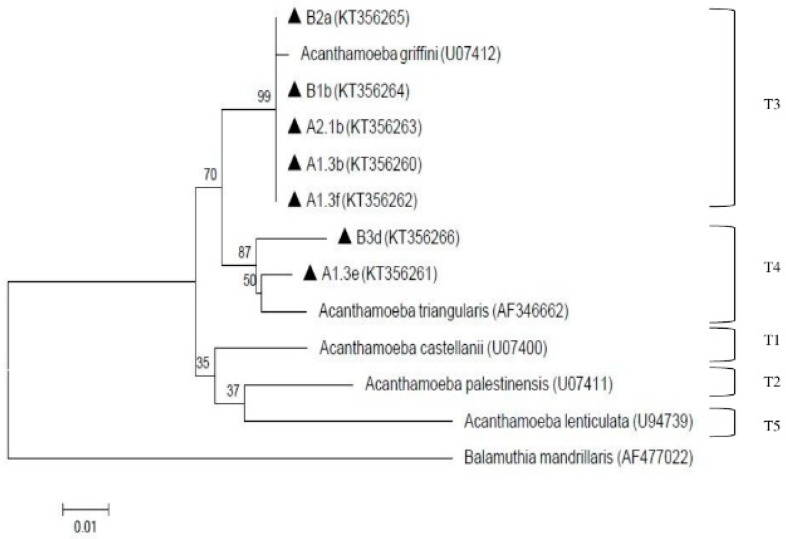
Neighbor-joining tree depicting the relationships between isolates and reference strains representing the genotypes of *Acanthamoeba*. The numbers on branch points depict the values of 1000 bootstrap replicates. GenBank accession numbers for reference sequences are indicated at the end of the species designations.

The presence of amoebae (*Acanthamoeba* and *Naegleria*) is shown in Table 1. Overall, both amoebae were detected qualitatively via cultivation and microscopy examination at all processing sites in both treatment plants. In order to decipher the specific species of *Acanthamoeba* and *Naegleria*, PCR analysis was further conducted. Eleven (11) *Acanthamoeba* isolates were selected for molecular analysis but only 7 were positive via PCR and were further found to be assembled under genotypes T3 and T4. Two isolates (A1.3b from pooled raw water of plant A and B1b from raw-1 of plant B) were under genotype T4 assemblage *Acanthamoeba triangularis (A. triangularis)* (AF346662) and five isolates (A1.3e and A1.3f from treated water of subdivision A-1; A2.1b from treated water of subdivision A-2; B2a from raw-2 of plant B; and B3d from raw-3 of plant B) were under genotype T3 with 99% identity with *Acanthamoeba griffini* (*A. griffini*) (U07412). These positive isolates (A1.3b, A1.3e, A1.3f, A2.1b, B1b, B2a, B3d) were deposited in GenBank with the respective accession numbers (KT356260, KT356261, KT356262, KT356263, KT356264, KT356265, KT356266). The result of the phylogenetic analysis is shown in Figure 3.

**Figure 4:**
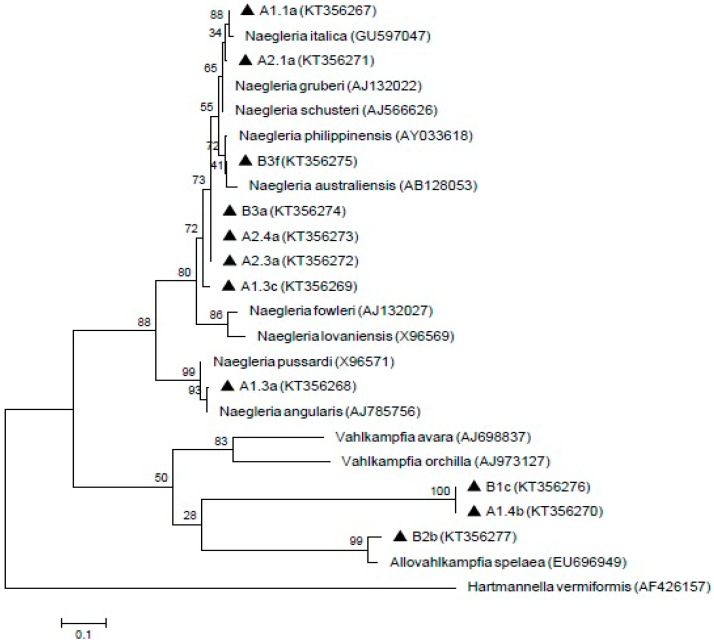
Maximum-likelihood tree depicting the relationships between isolated and reference strains of *Naegleria, Vahlkampfia* and *Allovahlkampfia*. The numbers on branch points depict the values of 1000 bootstrap replicates. GenBank accession numbers for reference sequences are indicated at the end of the species designations.

Meanwhile, 11 *Naegleria*-like isolates were found positive by using ITS1/ITS2 primer sets that amplified the 5.8S region. The isolate A1.1a was 100% homology with *Naegleria italica* (*N. italica*) (GU597047) and closely similar to A2.1a isolate. In addition, B3f formed a clade with *Naegleria australiensis* (*N. australiensis*) (AB128053) and showed 99% homology with *Naegleria philippinensis* (*N. philippinensis*) (AY033618). A1.3a isolate revealed 99% and 98% identity with *Naegleria angularis* (*N. angularis*) (AJ785756) and *Naegleria pussardi* (*N. pussardi*) (X96571), respectively. Meanwhile, A1.4b, B1c and B2b isolates formed a clade with *Vahlkampfia* group with B2b showing similarities with *Allovahlkampfia spelaea* (EU696949). However, the A1.3c, A2.3a, A2.4a and B3a isolates were not grouped with any reference sequences. The sequences of these isolates (A1.1a, A1.3a, A1.3c, A1.4b, A2.1a, A2.3a, A2.4a, B1c, B2b, B3a, and B3f) have been deposited in GenBank with the respective accession numbers (KT356267, KT356268, KT356269, KT356270, KT356271, KT356272, KT356273, KT356276, KT356277, KT356274, and KT356275). The result of the phylogenetic analysis is shown in Figure 4. 

### 3.5. Parasites Detected in the Distribution Systems

One (DS-1) out of seven randomly selected distribution system sites was found to be positive with *Cryptosporidium* oocyst (0.1 oocyst/L) as well as cultivable free-living amoebae, *Acanthamoeba* and *Naegleria* as shown in Table 1.

### 3.6. Fecal Coliform Counts

A total of 55 samples were collected for the isolation of fecal coliforms. Subdivision A-2 showed the highest presence of fecal coliforms (44.4%; 8/18) followed by subdivision A-1 (27.8%; 5/18). Meanwhile, 41.7% (5/12) were positive for fecal coliform counts in plant B while none were found in distribution system sites. 

The concentration of fecal coliform counts measured is shown in Table 3. In subdivision A-1, raw water showed the highest fecal coliform counts (13.33 ± 15.28 CFU/100 mL) in comparison to coagulation site (13.33 ± 5.77 CFU/100 mL) with the overall mean ± SD of 4.44 ± 8.56 CFU/100 mL (range of concentration: 0 to 30 CFU/100 mL). Fecal coliforms were not found in flocculation, sedimentation, filtration and treated water sites. Meanwhile, for subdivision A-2, raw water (26.00 ± 21.63 CFU/100 mL) also showed the highest fecal coliform counts in comparison to coagulation (13.33 ± 5.77 CFU/100 mL) and flocculation (10.00 ± 10.00 CFU/100 mL) sites. The water samples from sedimentation, filtration and treated water processing sites were found negative for fecal coliform counts. The overall mean ± SD for subdivision A-2 was 8.22 ± 12.94 CFU/100 mL with range of concentration: 0 to 50 CFU/100 mL. For plant B, raw-1 (15.00 ± 21.21 CFU/100 mL) site showed the highest fecal coliform counts followed by raw-3 (15.00 ± 7.07 CFU/100 mL) site. Both raw-2 and sedimentation sites revealed an equal count of fecal coliforms with 5.00 ± 7.07 CFU/100 mL each. However, fecal coliform was not detected in filtration and treated water sites. An overall mean ± SD of plant B revealed 6.67 ± 9.85 CFU/100 mL with concentrations ranging from 0 to 30 CFU/100 mL.

### 3.7. Physical and Chemical Parameters of Each Treatment Plant and the Distribution Systems

The physical and chemical parameters are summarized in Table 4. The parameters (overall mean for plant A and B) included temperature (overall mean: 26.17 ± 1.15 °C), conductivity (overall mean: 0.063 ± 0.027 ms/cm), TDS (overall mean: 0.045 ± 0.019 g/L), salinity (overall mean: 0.030 ± 0.012 ppt), DO (overall mean: 3.245 ± 0.867%), pH (overall mean: 6.12 ± 0.26 pH), ORP (overall mean: 44.123 ± 16.188 mv) and turbidity (overall mean: 13.66 ± 16.743 NTU (Nephelometric Turbidity Unit)). Meanwhile, chemical tests including ammonia (overall mean: 0.209 ± 0.112 mg/L), chlorine (overall mean: 0.322 ± 0.680 mg/L), nitrite (overall mean: 0.010 ± 0.030 mg/L), nitrate (overall mean: 0.039 ± 0.057 mg/L) and fluoride (overall mean: 0.281 ± 0.163 mg/L) were also measured and analyzed. In addition, physical and chemical parameters were also measured from the samples taken from the distribution systems. Plant B was found to have lower overall mean values for ammonia, chlorine, nitrate and fluoride in comparison with plant A and the distribution systems. Plant B also showed lower overall mean for turbidity. Overall, the readings for physical and chemical parameters were in similar ranges between both treatment plants and distribution system sites.

### 3.8. Statistical Analysis

The results of statistical analysis of the association between physical, chemical and biological parameters and the occurrence of *Giardia* cysts and *Cryptosporidium* oocysts are presented in Table 5. Significant positive correlations were observed between the presence of *Giardia* cysts with fluoride (*r* = 0.611, *p* < 0.01) and fecal coliforms (*r* = 0.855, *p* < 0.01). Similarly, the occurrence of *Cryptosporidium* oocysts showed significant correlations with fluoride (*r* = 0.478, *p* < 0.05) and fecal coliforms (*r* = 0.536, *p* < 0.05). No significant correlations were found between the occurrence of these protozoa and the physical parameters. Moreover, there was also no significant correlation with ammonia, chlorine, nitrate and nitrite.

## 4. Discussion

Drinking water treatment plant plays a key role in providing safe drinking water to consumers and the findings from this study will give us an insight on the water quality as well as processing sites that needs regular monitoring and intervention steps to be taken. Raw water is easily contaminated, thus, drinking water need to be processed in the treatment plant before being distributed. Coagulation and flocculation process is considered an essential component in a treatment plant to assist filtration process to function effectively, hence, able to remove microorganisms as well as other solid matters caused by soil erosion or decaying vegetation [36]. Finished water is then placed in a closed tank or reservoir before being distributed. Nonetheless, the multi-barrier system is considered to be the best option to reduce waterborne pathogens in drinking water. However, even though the treatment plant is equipped with proper facilities and technologies, microorganisms may still be able to penetrate through these treatment processes. 

### 4.1. Occurrence of (Oo)cysts and Other Parasites

From this study, the overall occurrence of *Giardia* cysts (32.9%) was shown to be higher compared to *Cryptosporidium* oocysts (18.8%). A similar study was conducted in three water treatment plants in Negeri Sembilan, Malaysia by Tan [25] who reported that out of 31 raw water samples, 16 samples were positive for *Giardia* cysts (range = 0 to 12 cysts/L) while 6 samples were found to be positive with *Cryptosporidium* oocysts (range = 0 to 5 oocysts/L). However, neither cysts nor oocysts were detected in treated water samples. These findings also coincided with an earlier study done in another state (i.e., Selangor) in Malaysia by Ahmad et al. [24]. They reported that *Giardia* cysts were detected in 90% of the raw water samples from two treatment plants while *Cryptosporidium* oocysts were not detected either in raw or treated water. Razzolini et al. [37] also reported that *Giardia* cysts were ubiquitous and more frequently detected compared to *Cryptosporidium* oocysts. 

While larger, *Giardia* cysts were more frequently found throughout this study compared to *Cryptosporidium* oocysts. Furthermore, most cases in other countries also revealed *Giardia* being more prevalent than *Cryptosporidium* [38,39,40]. The higher occurrence of *Giardia* in raw water indicates the higher cyst contamination of the river. Moreover, the lower numbers of *Cryptosporidium* oocysts detected throughout various sites may indicate a lesser extent of contamination from human and animal sources. A previous study done by Lee et al. [41] from five rivers that flow along five different indigenous villages (Kuala Pangsun, Bentong, Kemensah, Pos Piah and Paya Lebar villages), showed a high occurrence of *Giardia* cysts (51.3%; 20/39) in comparison to *Cryptosporidium* oocysts (23.1%; 9/39). Another study done by Lim and Ahmad [42] also reported *Giardia* positive in 67% of samples while only 6% was found to be positive with *Cryptosporidium* oocyst. Hence, there might be an overload throughout the treatment processes or process failure that has enabled cyst to penetrate into the treated water. 

With regards to the treated water in the present study, *Giardia* cysts were found in one treated water sample each of subdivision A-2 (0.02 ± 0.04 cysts/L) and plant B (0.13 ± 0.23 cysts/L) while *Cryptosporidium* oocysts were not detected. In subdivision A-1, both parasitic protozoa were not found in filtration and treated water. However, in subdivision A-2, *Giardia* cysts was found in treated but not in filtration process. *Giardia* cysts were also found throughout the processes in plant B. This may be due to the simpler processes that the smaller plant B utilizes which does not incorporate the coagulation and flocculation processes. A similar study conducted in Scotland by Smith et al. [43] reported the presence of *Giardia* cysts in treated water. Furthermore, another study carried out in Northern Spain revealed the occurrence of *Giardia* and *Cryptosporidium* in both raw and treated water samples obtained from treatment plants that only include rapid filtration and disinfection processes while no (oo)cysts were found in treated water samples from conventional treatment facilities [44]. These data further corroborate our findings that minimal treatment processes involved are insufficient in removing protozoa. Thus, without comprehensive treatment, parasites and other microbial contaminants can more easily penetrate into sedimentation and filtration sites due to their sizes. States et al. [45] had also reported that small numbers of protozoa can also be found in finished water despite the absence of treatment failures.

Based on the observation of this study, the presence of both *Giardia* and *Cryptosporidium* is most likely to be associated with the presence of human settlements, agricultural farm, sand mining activity and other activities that were carried out by the villagers such as swimming or garbage disposal throughout the river. There was also an animal farm located along the river that acts as the main source of raw water for one of the drinking water treatment plants. As cattle were seen grazing freely, there is a potential that infected animals may act as sources of parasites contamination into the river water via animal defecation through washed-off during wet season. In addition, although these animals are provided with drinking water in the cattle shed, as they roam freely, uninfected animals may also be exposed to drinking contaminated water from the river. This finding is supported by a previous study done by Farizawati et al. [46] on the role of livestock farms as one of the factors that contribute to contamination of (oo)cysts into the river at the Langat Basin, Selangor, Malaysia. The study showed a slightly higher number of *Giardia* cysts (16.4%; 11/67) compared to *Cryptosporidium* oocysts (14.9%; 10/67) in the river water samples adjacent to cattle farms. In addition, different *Giardia* and *Cryptosporidium* values in raw water samples from subdivisions A-1 and A-2 were observed, although both were from the same source. This is because the water flows into separate weirs before entering the two modules of the treatment plant. Subdivision A-2 revealed a higher occurrence of both (oo)cysts. Based on our observation, the weirs that function as the “entering point” of raw water at subdivision A-2 was located nearer to the farm compared to subdivision A-1. Thus, the possibility of fecal contamination from the animals may contribute to the higher occurrence of (oo)cysts. However, the present study did not include the examination of cattle feces due to logistic constraint. This aspect will need to be incorporated in future study for a better understanding of the parasite transmission and possible environmental contamination. 

Other parasites that were also detected include *Spirometra* ova-like, *Blastocystis*-like, nematode larvae-like and *Taenia* ova-like. These parasites were found throughout the plant except for treated water and distribution system sites. A study in Turkey has shown the presence of helminth ova or eggs from river water samples as reported by Bakir et al. [47]. On the other hand, a recent report conducted in Iraq, revealed drinking water samples were contaminated with various parasites such as *E. coli*, *Giardia lamblia* and hookworm [48]. The fecal discharge from both infected humans and/or animals might be the factor that causes the contamination. Brooker [49] also considered climate and topography as another aspect that may influence the survival and distribution of these helminthes. 

### 4.2. Occurrence of Free-Living Amoebae

Meanwhile, the presence of free-living amoebae (FLA) was detected at almost all sites of the treatment plant. This is due to their wide ecological distribution in the environment [50] as well as being naturally present within source water and their ability to survive treatment processes [51]. FLA have been recovered from various domestic water systems, such as tap water [52], cooling towers [53], swimming pools [31,54], hydrotheraphy baths [55] and hospital water networks [56,57]. Environmental factors, including rain and wind, may influence the circulation of cysts in the environment. The presence of algae on the treatment plant walls may also enhance the growth of bacteria by providing food and resulted in the colonization of FLA. Both FLA, coprophilic organisms [58], can grow at room temperature followed by 37 °C, thus, treatment plants are considered favorable sites for FLA growth. Due to the FLA’s wide distribution, isolates from selected sampling sites (raw water, treated water and distribution system) were subjected for further molecular analysis. The reference strains taken from the National Center for Biotechnology Information (NCBI) showed similarities towards all 18 isolates. 

To date, *Acanthamoeba* have been classified up-to-date into 20 different genotypes (T1–T20) with T20 being recovered from internal organs of a dead toucan (initially assigned to T4) as reported by Schuster and Visvesvara [59], Visvesvara et al. [60] and Fuerst et al. [61]. Genotype T3, which belongs to the same cluster as *Acanthamoeba griffini*, was previously considered as non-pathogenic, but was reported to be association with keratitis infection [62,63,64,65,66,67]. However, the infections caused by these genotypes are considered rare [68,69,70]. Meanwhile, genotype T4 was considered to be predominant for both clinical and environmental specimens as stated by Booton et al. [71]. However, it is still unknown whether T4 isolates produce keratitis due to greater virulence or prevalence [66]. From this study, genotype T3 and T4 were detected from the water samples. The presence of *Acanthamoeba* in finished water can be attributed to its resistance of cyst stage towards chlorine as chlorine has been reported unable to eliminate *Acanthamoeba* in the doses that were commonly used in water treatment [50,72]. The possibility of the pathogenic strains contaminating the water used by consumers must not be taken lightly by the authorities. Thus, several precautions must be adhered such as avoiding water-related activities such as swimming in the river when having eye injury or accidental squirt into the nasal cavity when bathing. Similarly, a previous study reported pathogenic genotypes belonging to T3, T4, T5 and T11 that were isolated from tap waters of tourist attraction sites in southern Iran [73]. Based on the findings from both previous and current studies, genotype T4 was generally detected in most of the samples, hence, T4 is considered most ubiquitous genotype in environmental sources [74]. 

Subsequently, the ITS primer set was reported to produce various amplicons that showed homology with *Naegleria*, *Hartmannella, Vahlkampfia* and *Willaertia* [34]. *Naegleria australiensis* and *Naegleria philippinensis* as being pathogenic in mice and may pose a potential threat to human health [75]. *Naegleria italica* was also shown to be pathogenic in experimental animals but, no human cases has been recorded thus far [76]. The results from the phylogenetic tree in the present study suggested that the isolates of B3a, A2.4a, A2.3a and A1.3c are not represented by any known *Naegleria* species. Hence, the relationships between the isolates and reference sequences are unknown and can possibly be new species. In our study, PCR amplification by using specific primers for the pathogenic strain, *Naegleria fowleri* (*N. fowleri*), did not produce any PCR products, thus, *N. fowleri* was not detected from these samples. Therefore, water from both of these treatment plants is safe from pathogenic *N. fowleri*. The result from this study supported the previous finding from Malaysian aquatic environments that there was absence of pathogenic *N. fowleri* and presence of several *Naegleria* species such as *Naegleria philippinensis* and *Naegleria schusteri* [77]. Hence, to date, Malaysian aquatic environment is still considered safe from *N. fowleri* infection. 

From this study, FLA was also observed in treated water and our findings were supported by a study done by Thomas and Ashbolt [58] that reported the presence of FLA in the effluents of treatment plants could be due not only to their resistance to the treatments but also their ability to continue growing in the different conduits of the plants, probably sheltered in biofilms. 

### 4.3. Parasites Detected in Distribution System Site

In addition, *Cryptosporidium, Acanthamoeba* and *Naegleria* were found positive in one of the distribution system (DS-1) sites situated in the vicinity of plant A compared to other sites that are located at a distance of more than 5 km from the treatment plants. The occurrence of these parasites may be due to the malfunction of the treatment processes or occurrence of post-treatment contamination. Factors such as leaking pipes, valves, joints and seals as well as contamination of the tap by the final users may be some possible reasons for the contamination [18]. Failure in the filtration stage or inadequate chlorine concentration below the minimal level may also contribute to the detection of parasites in the finished water. Therefore, optimal disinfection with chlorine is a very important option to prevent the transmission of these parasites. 

### 4.4. Fecal Coliform Count

Meanwhile, fecal coliform count was also carried out as a preferred method and basis indicator that assists in assessing water quality. The excretion of feces into the river water is considered the biggest concern especially to water authorities as it can cause diseases such as gastroenteritis. Previous findings showed the ability of coliform bacteria to grow within drinking water systems [78,79]. Hence, it is considered a significant contributor to the biofilm populations, even though it does not necessarily present health risk to the consumers. From this study, a higher number of fecal coliform was detected in raw water in comparison to other processing sites due to raw water being closely related to both humans and animals. However, fecal coliform was not found in treated water and distribution system sites, thus highlighting that the treatment plant is efficient in eliminating fecal contamination.

### 4.5. Statistical Analysis 

Statistical analysis showed positive correlations were observed between presence of *Giardia* and *Cryptosporidium* (oo)cysts with fluoride and fecal coliforms. Fluoride is added into drinking water during disinfection process but high content of fluoride may have its own affect towards our health [80,81]. However, little is known regarding the association of fluoride with *Giardia* and *Cryptosporidium* (oo)cysts. Meanwhile, fecal coliform count is usually carried out together with other water-related studies as it indicates the presence of potential pathogenic microbes in water that are commonly transmitted via fecal–oral route. A similar study done by [82] reported a correlation between fecal coliforms and *Cryptosporidium* obtained from drinking water from district of Ludhiana, India. In contrast, other studies done by Lee et al. [41] and Azman et al. [83] have reported no significant correlation between the parameters and protozoa, hence, the parameters were considered not suitable to be used as indicators. 

## 5. Conclusions

In summary, these waterborne parasites were detected frequently in raw water followed by coagulation, flocculation, sedimentation and filtration sites. This research study illustrated the need for better monitoring of drinking water quality especially in drinking water systems that may pose health threat to the consumers. This is vital as the results of the study demonstrated that protozoan parasites and free-living amoebae occur in various treatment processes sites. For future study, it is crucial to incorporate recovery efficiency measurements and quantitative microbial risk assessment. These data will provide beneficial information to assist key stakeholders in formulating appropriate monitoring and control strategies to mitigate the contamination for improved public water quality systems.

## Figures and Tables

**Table 1:** Overall occurrence of parasites detected at each drinking water treatment plant and the distribution systems.

Location	Parasites							
	*Giardia*		*Cryptosporidium*		Other Parasites		Amoebae	
	+/N	%	+/N	%	+/N	%	+/N	%
Subdivision A-1	6/30	20.0	5/30	16.7	12/30	40.0	18/18	100.0
Subdivision A-2	13/30	43.3	6/30	20.0	9/30	30.0	18/18	100.0
Plant B	9/18	50.0	4/18	22.2	6/18	3.3	18/18	100.0
DS	0/7	0	1/7	14.3	0/7	0	1/7	14.3
Total Positive	28/85	32.9	16/85	18.8	27/85	31.8	55/61	90.2

DS = Distribution system; + = positive samples, N = total of samples; % = percentage.

**Table 2:** Concentration of *Giardia* cysts and *Cryptosporidium* oocysts at each treatment plant.

**Plant A**			
**Location**	**Site**	***Giardia* (cysts/L) ^a^**	***Cryptosporidium* (oocysts/L) ^a^**
Subdivision A-1	Raw	0.18 ± 0.35	0.02 ± 0.04
	Coagulation	0.06 ± 0.05	0.04 ± 0.09
	Flocculation	0.02 ± 0.04	0.02 ± 0.04
	Sedimentation	0	0.06 ± 0.09
	Filtration	0	0
	Treated	0	0
	**Overall mean ± SD**	0.04 ± 0.15	0.02 ± 0.06
	**Range of concentration**	0–0.80	0–0.20
Subdivision A-2	Raw	0.44 ± 0.53	0.04 ± 0.05
	Coagulation	0.14 ± 0.11	0.14 ± 0.19
	Flocculation	0.18 ± 0.20	0.04 ± 0.05
	Sedimentation	0.06 ± 0.13	0
	Filtration	0	0
	Treated	0.02 ± 0.04	0
	**Overall mean ± SD**	0.14 ± 0.27	0.04 ± 0.09
	**Range of concentration**	0–1.20	0–0.40
**Plant B**			
**Location**	**Site**	***Giardia* (cysts/L) ^a^**	***Cryptosporidium* (oocysts/L) ^a^**
Plant B	Raw-1	0.17 ± 0.29	0.03 ± 0.06
	Raw-2	0.17 ± 0.12	0
	Raw-3	0.17 ± 0.21	0.07 ± 0.06
	Sedimentation	0.03 ± 0.06	0.03 ± 0.06
	Filtration	0.03 ± 0.06	0
	Treated	0.13 ± 0.23	0
	**Overall mean ± SD**	0.12 ± 0.17	0.02 ± 0.04
	**Range of concentration**	0–0.50	0–0.10

^a^ Mean ± SD of each drinking water treatment plant processing sites. SD = Standard deviation; L = liter.

**Table 3:** Concentration of fecal coliform counts at various processing sites in each treatment plant.

**Plant A**		
**Location**	**Site**	**Fecal Coliforms (CFU/100 mL) (Mean ± SD)**
Subdivision A-1	Raw	13.33 ± 15.28
	Coagulation	13.33 ± 5.77
	Flocculation	0
	Sedimentation	0
	Filtration	0
	Treated	0
	Overall mean ± SD	4.44 ± 8.56
	Range of concentration	0–30
Subdivision A-2	Raw	26.00 ± 21.63
	Coagulation	13.33 ± 5.77
	Flocculation	10.00 ± 10.00
	Sedimentation	0
	Filtration	0
	Treated	0
	Overall mean ± SD	8.22 ± 12.94
	Range of concentration	0–50
**Plant B**		
**Location**	**Site**	**Fecal coliforms (CFU/100 mL) (Mean ± SD)**
Plant B	Raw-1	15.00 ± 21.21
	Raw-2	5.00 ± 7.07
	Raw-3	15.00 ± 7.07
	Sedimentation	5.00 ± 7.07
	Filtration	0
	Treated	0
	Overall mean ± SD	6.67 ± 9.85
	Range of concentration	0–30

**Table 4:** Physical and chemical parameters of each treatment plant and the distribution systems.

Parameter	Subdivision A-1 ^a^	Subdivision A-2 ^a^	Plant B ^a^	DS ^a^
Physical				
Temperature (°C)	25.41 ± 0.99	26.32 ± 0.93	27.19 ± 0.84	26.55 ± 0.75
Conductivity (ms/cm)	0.076 ± 0.016	0.075 ± 0.015	0.021 ± 0.010	0.051 ± 0.022
Total dissolved solids (TDS g/L)	0.050 ± 0.010	0.050 ± 0.013	0.030 ± 0.027	0.047 ± 0.018
SAL (ppt)	0.030 ± 0.007	0.030 ± 0.008	0.010 ± 0.008	0.020 ± 0.011
Dissolved oxygen (%)	3.543 ± 0.604	3.507 ± 0.804	2.300 ± 0.692	2.65 ± 0.292
pH	6.11 ± 0.363	6.14 ± 0.211	6.12 ± 0.022	6.13 ± 0.261
ORP (mv)	50.73 ± 22.31	39.56 ± 11.12	40.72 ± 1.12	29.06 ± 0.959
Turbidity (NTU)	18.19 ± 21.55	16.57 ± 12.39	1.25 ± 1.34	0.673 ± 0.403
Chemical				
Ammonia (NH_3_, mg/L)	0.224 ± 0.102	0.216 ± 0.122	0.174 ± 0.111	0.211 ± 0.089
Chlorine (CI_2_, mg/L)	0.310 ± 0.647	0.356 ± 0.763	0.286 ± 0.620	0.963 ± 0.296
Nitrite (NO^2−^, mg/L)	0.019 ± 0.047	0.004 ± 0.005	0.005 ± 0.006	0.003 ± 0.001
Nitrate (NO^3−^, mg/L)	0.061 ± 0.085	0.028 ± 0.022	0.022 ± 0.016	0.099 ± 0.021
Fluoride (F^−^, mg/L)	0.301 ± 0.188	0.288 ± 0.161	0.237 ± 0.117	0.250 ± 0.059

SAL: salinity; ORP: oxidation reduction potential. ^a^ Mean ± SD of each drinking water treatment plants and distribution system.

**Table 5:** Correlation analysis between *Giardia* and *Cryptosporidium* with physical, chemical and biological parameters.

Parameter	*Giardia*	*Cryptosporidium*
Physical		
Temperature, °C	0.133	0.193
Conductivity, ms/cm	−0.158	0.033
Total dissolved solids (g/L)	−0.083	0.088
SAL (ppt)	−0.283	−0.138
Dissolved oxygen (%)	−0.36	−0.117
pH	0.232	0.111
ORP (mv)	−0.288	0.056
Turbidity (NTU)	0.333	0.36
Chemical		
Ammonia (NH_3_, mg/L)	−0.052	0.169
Chlorine (Cl_2_, mg/L)	−0.225	−0.351
Nitrate (NO^3^⁻, mg/L)	−0.105	−0.115
Nitrite (NO^2^⁻, mg/L)	−0.197	0.015
Fluoride (F⁻, mg/L)	0.611 **	0.478 *
Biological		
Fecal coliforms (CFU/100 mL)	0.855 **	0.536 *

* Correlation is significant at the 0.05 level. ** Correlation is significant at the 0.01 level.

## References

[1] WHO/UNICEF Joint Statement Clinical Management of Acute Diarrhoea, 2004. Geneva and New York: World Health Organization, Department of Child and Adolescent Health and Development, and United Nations Children’s Fund, Programme Division (WHO/FCH/CAH/04.07). apps.who.int/iris/bitstream/10665/68627/1/WHO_FCH_CAH_04.7.pdf.

[2] Young M., Wolfheim C., Marsh D.R., Hammamy D. (2012). World Health Organization/United Nations Children’s Fund Joint Statement on Integrated Community Case Management: An Enquity-Focused Strategy to Improve Access to Essential Treatment Services for Children. Am. J. Trop. Med. Hyg..

[3] Clasen T., Bastable A. (2003). Fecal contamination of drinking water during collection and household storage: The need to extend protection to the point of use. J. Water Health.

[4] Geldreich E.E., Geldreich E.E. (1996). Waterborne pathogen invasions: A case for water quality protection in distribution. Microbial Quality of Water Supply in Distribution Systems.

[5] Nagdeve D.A. (2003). Population and land use in Maharashtra. Bhartiya Samajik Chintan Kolkata.

[6] Butchart S.H.M., Stattersfield A.J., Baillie J., Bennun L.A., Stuart S.N., Akcakaya H.R., Hilton-Taylor C., Mace G.M. (2005). Using Red List Indices to measure progress towards the 2010 target and beyond. Philos. Trans. R. Soc..

[7] Khatri K.B., Vairavamoorthy K. (2007). Challenges for Urban Water and Sanitation in the Developing Countries.

[8] Karanis P., Kourenti C., Smith H. (2007). Waterborne transmission of protozoan parasites: A worldwide review of outbreaks and lessons learnt. J. Water Health.

[9] Fayer R., Morgan U., Upton S.J. (2000). Epidemiology of *Cryptosporidium*: Transmission, detection and identification. Int. J. Parasitol..

[10] Slifko T.R., Smith H.V., Rose J.B. (2000). Emerging parasite zoonoses associated with water and food. Int. J. Parasitol..

[11] Thompson R.C.A. (2000). Giardiasis as a re-emerging infectious disease and its zoonotic potential. Int. J. Parasitol..

[12] Coupe C., Delabre K., Pouillot R., Houdart S., Santillana-Hayat M., Derouin F. (2006). Detection of *Cryptosporidium*, *Giardia* and *Enterocytozoon bieneusi* in surface water, including recreational areas: A one-year prospective study. FEMS Immunol. Med. Microbiol..

[13] Graczyk T., Sunderland D., Awantang G., Mashinski Y., Lucy F., Graczyk Z., Chomicz L., Breysse P. (2010). Relationships among bather density, levels of human waterborne pathogens, and fecal coliform counts in marine recreational beach water. Parasitol. Res..

[14] Mason B.W., Chalmers R.M., Carnicer-Pont D., Casemore D.P. (2010). A *Cryptosporidium hominis* outbreak in north-west Wales associated with low oocyst counts in treated drinking water. J. Water Health.

[15] Yoder J., Beach M. (2010). *Cryptosporidium* surveillance and risk factors in the United States. Exp. Parasitol..

[16] Webb L.M. (2015). Giardiasis Outbreak Associated with Travel to Mexico—Kansas. http://www.kdheks.gov/epi/download/KS_FEB15_Mexico_Giardia.pdf.

[17] Gertler M., Durr M., Renner P., Poppert S., Askar M., Breidenbach J., Frank C., Preußel K., Schielke A., Werber D. (2015). Outbreak of *Cryptosporidium hominis* following river flooding in the city of Halle (Saale), Germany, August 2013. BMC Infect. Dis..

[18] Amer A.S. (2012). Monitoring for the presence of parasitic protozoa and free-living amoebae in drinking water plants. J. Nat. Res. Dev..

[19] Trabelsi H., Dendana F., Sellami A., Sellami H., Cheikhrouhou F., Neji S., Makni F., Ayadi A. (2012). Pathogenic free-living amoebae: Epidemiology and clinical review. Pathol. Biol..

[20] Por Y.M., Mehta J.S., Chua J.L.L., Koh T.-H., Khor W.B., Fong A.C.Y., Lim J.W.K., Heng W.J., Loh R.S.K., Lim L. (2009). *Acanthamoeba* keratitis associated with contact lens wear in Singapore. Am. J. Ophthalmol..

[21] Verani J.R., Lorick S.A., Yoder J.S., Beach M.J., Braden C.R., Roberts J.M., Conover C.S., Chen S., McConnell K.A., Chang D.C. (2009). National outbreak of *Acanthamoeba* keratitis associated with use of a contact lens solution, United States. Emerg. Infect. Dis..

[22] Marciano-Cabral F., Cabral G. (2003). *Acanthamoeba* spp. as agents of disease in humans. Clin. Microbiol. Rev..

[23] Shakoor S., Beg M.A., Mahmood S.F., Bandea R., Sriram R., Noman F., Ali F., Visvesvara G.A., Zafar A. (2011). Primary amebic meningoencephalitis caused by *Naegleria fowleri*, Karachi, Pakistan. Emerg. Infect. Dis..

[24] Ahmad R.A., Lee E., Tan I.T.L., Mohamad-Kamel A.G. (1997). Occurrence of *Giardia* cysts and *Cryptosporidium* oocysts in raw and treated water from two water treatment plants in Selangor, Malaysia. Water Res..

[25] Tan I.T.L. (1997). Pengesanan Protozoa Pathogen di Tiga Buah Loji Pembersihan Air di Negeri Sembilan. Master’s Thesis.

[26] State Government of Sarawak 2012 Publication, Sarawak: Facts and Figures. http://www.sarawak.gov.my/ebook/Fact_and_Figures_2012.

[27] Department of Statistics Malaysia (2010). Population and Housing Census of Malaysia.

[28] Kuok K.K., Sobri H., Po-Chan C. (2011). A review of integrated river basin management for Sarawak River. Am. J. Environ. Sci..

[29] KWB (Kuching Water Board) Annual Report Year 2014. http://www.kwb.gov.my/modules/web/pages.php?mod=publication&sub=publication_show&id=2.

[30] USEPA (United States Environmental Protection Agency) 2012 Method 1623.1: *Cryptosporidium* and *Giardia* in Water by Filtration/IMS.FA. http://water.epa.gov/scitech/drinkingwater/labcert/upload/epa816r12001.pdf.

[31] Init I., Lau Y.L., Arine A.F., Foead A.I., Neilson R.S., Nissapatorn V. (2010). Detection of free-living amoebae, *Acanthamoeba* and *Naegleria*, in swimming pools, Malaysia. Trop. Biomed..

[32] Schroeder J.M., Booton G.C., Hay J., Niszl I.A., Seal D.V., Markus M.B., Fuerst P.A., Byers T.J. (2001). Use of subgenic 18S ribosomal DNA PCR and sequencing for genus and genotype identification of *Acanthamoebae* from humans with keratitis and from sewage sludge. J. Clin. Microbiol..

[33] Pelandakis M., Pernin P. (2002). Use of multiplex PCR and PCR restriction enzyme analysis for detection and exploration of the variability in the free-living amoeba *Naegleria* in the environment. Appl. Environ. Microbiol..

[34] Altschul S.F., Gish W., Miller W., Myers E.W., Lipman D.J. (1990). Basic local alignment search tool. J. Mol. Biol..

[35] Tamura K., Stecher G., Peterson D., Filipski A., Kumar S. (2013). MEGA6: Molecular evolutionary genetics analysis version 6.0. Mol. Biol. Evol..

[36] Helmi M.J. (2005). Experimental Study on the Factor Affecting Coagulation and Flocculation. Bachelor’s Thesis.

[37] Razzolini M.T.P., da Silva Santos T.F., Bastos V.K. (2010). Detection of *Giardia* and *Cryptosporidium* cysts/oocysts in watersheds and drinking water sources in Brazil urban areas. J. Water Health.

[38] Horman A., Rimhanen-Finne R., Maunula L., von Bonsdorff C.H., Torvela N., Heikinheimo A., Hanninen M.L. (2004). *Campylobacter* spp., *Giardia* spp., *Cryptosporidium* spp., noroviruses, and indicator organisms in surface water in southwestern Finland, 2000–2001. Appl. Environ. Microbiol..

[39] Rimhanen-Finne R., Vuorinen A., Marmo S., Malmberg S., Hanninen M.-L. (2004). Comparative analysis of *Cryptosporidium*, *Giardia* and indicator bacteria during sewage sludge hygienization. Lett. Appl. Microbiol..

[40] Brianesco R., Bonadonna L. (2005). An Italian study of *Cryptosporidium* and *Giardia* in wastewater, fresh water and treated water. Environ. Monit. Asses..

[41] Lee S.C., Ngui R., Tan T.K., Muhammad Aidil R., Init I., Lim Y.A.L. (2014). Aquatic biomonitoring of *Giardia* cysts and *Cryptosporidium* oocysts in Peninsular Malaysia. Environ. Sci. Pollut. Res..

[42] Lim Y.A.L., Ahmad R.A. (2004). Occurrence of *Giardia* cysts and *Cryptosporidium* oocysts in the Temuan Orang Asli (Aborigine) river system. Southeast Asian J. Trop. Med..

[43] Smith H.V., Robertson L.J., Gilmour R.A., Morris G.P., Girdwood R.W.A., Smith P.G. (1993). The occurrence and viability of *Giardia* cysts in Scottish raw and final waters. Water Environ. J..

[44] Carmena D., Aguinagalde X., Zigorraga C., Fernandez-Crespo J.C., Ocio J.A. (2007). Presence of *Giardia* cysts and *Cryptosporidium* oocysts in drinking water supplies in northern Spain. J. Appl. Microbiol..

[45] States S.M., Stadterman K., Ammon L., Vogel P., Baldizar J., Wright D., Conley L., Sykora J. (1997). Protozoa in river water: Sources, occurrence, and treatment. J. AWWA.

[46] Farizawati S., Lim Y.A.L., Ahmad R.A., Fatimah C.T.N.I., Siti-Nor Y. (2005). Contribution of cattle farms towards river contamination with *Giardia* cysts and *Cryptosporidium* oocysts in Sungai Langat Basin. Trop. Biomed..

[47] Bakir B., Tanyuksel M., Saylam F., Tanriverdi S., Araz R.E., Hacim A.K., Hasde M. (2003). Investigation of waterborne parasites in drinking water sources of Ankara, Turkey. J. Microbiol..

[48] Al-Morshidy K.A.H., Al-Amari M.J.Y. (2015). Detection of parasitic contamination in Hilla city drinking water/Babylon province/Iraq. Adv. Nat. Appl. Sci..

[49] Brooker S. (2007). Spatial epidemiology of human schistosomiasis in Africa: Risk models, transmission dynamics and control. Trans. R. Soc. Trop. Med. Hyg..

[50] Khan N.A. (2006). *Acanthamoeba*: Biology and increasing importance in human health. FEMS Microbiol. Rev..

[51] Hoffmann R., Michel R. (2001). Distribution of free-living amoebae (FLA) during preparation and supply of drinking water. Int. J. Hyg. Environ. Health.

[52] Coskun K.A., Ozcelik S., Tutar L., Elaldi N., Tutar Y. (2013). Isolation and identification of free-living amoebae from tap water in Sivas, Turkey. BioMed. Res. Int..

[53] Scheikl U., Tsao H.-F., Horn M., Indra A., Walochnik J.F. (2016). Free-living amoebae and their associated bacteria in Austrian cooling towers: A 1-year routine screening. Parasitol. Res..

[54] Leonska-Duniec A., Adamska M., Skotarczak B. (2015). Molecular identification of free-living amoebae isolated from artificial water bodies located in Poland. Acta Protozool..

[55] Hassan A., Farouk H., Hassanein F., Abdul-Ghani R., Abdelhady A.H. (2012). *Acanthamoeba* contamination of hemodialysis and dental units in Alexandria, Egypt: A neglected potential source of infection. J. Infect. Public Health.

[56] Thomas V.K., Herrera-Rimann K., Blanc D.S., Greub G. (2006). Biodiversity of amoebae and amoebae-resisting bacteria in a hospital water network. Appl. Environ. Microbiol..

[57] Muchesa P., Barnard T.G., Bartie C. (2015). The prevalence of free-living amoebae in a South African hospital water distribution system. S. Afr. J. Sci..

[58] Thomas J.M., Ashbolt N.J. (2011). Do free-living amoebae in treated drinking water systems present an emerging health risk?. Environ. Sci. Technol..

[59] Schuster F.L., Visvesvara G.S. (2004). Free-living amoebae as opportunistic and non-opportunistic pathogens of humans and animals. Int. J. Parasitol..

[60] Visvesvara G.S., Moura H., Schuster F.L. (2007). Pathogenic and opportunistic free-living amoebae: *Acanthamoeba* spp., *Balamuthia mandrillaris*, *Naegleria fowleri*, and *Sappinia diploidea*. FEMS Immunol. Med. Microbiol..

[61] Fuerst P.A., Booton G.C., Crary M. (2015). Phylogenetic analysis and the evolution of the 18S rRNA gene typing system of *Acanthamoeba*. J. Eukaryot. Microbiol..

[62] Ledee D.R., Hay J., Byers T.J., Seal D.V., Kirkness C.M. (1996). *Acanthamoeba griffin*i. Molecular characterization of a new corneal pathogen. Investig. Opthalmol. Vis. Sci..

[63] Stothard D.R., Schroeder-Diedrich J.M., Awwad M.H., Gast R.J., Ledee D.R., Rodriguez-Zaragoza S., Dean C.L., Fuerst P.A., Byers T.J. (1998). The evolutionary history of the genus *Acanthamoeba* and the identification of eight new 18S rRNA gene sequence types. J. Eukaryot. Microbiol..

[64] Walochnik J., Haller-Schober E.M., Kolli H., Picher O., Obwaller A., Aspock H. (2000). Discrimination between clinically relevant and nonrelevant *Acanthamoeba* strains isolated from contact lens wearing keratitis patients in Austria. J. Clin. Microbiol..

[65] Booton G.C., Kelly D.J., Chu Y.W., Seal D.V., Houang E., Lam D.S., Byers T.J., Fuerst P.A. (2002). 18S ribosomal DNA typing and tracking of *Acanthamoeba* species isolates from corneal scrape specimens, contact lenses, lens cases and home water supplies of *Acanthamoeba* keratitis patients in Hong Kong. J. Clin. Microbiol..

[66] Khan N.A., Jarroll E.L., Paget T.A. (2002). Molecular and physiological differentiation between pathogenic and non-pathogenic *Acanthamoeba*. Curr. Microbiol..

[67] De Jonckheere J.F. (2003). Epidemiological typing of *Acanthamoeba* strains isolated from keratitis cases in Belgium. Bull. Soc. Belge Ophtalmol..

[68] Maghsood A.H., Sissons J., Rezaian M., Nolder D., Warhurst D., Khan N.A. (2005). *Acanthamoeba* genotype T4 from the UK and Iran and isolation of the T2 genotype from clinical isolates. J. Med. Microbiol..

[69] Ledee D.R., Iovieno A., Miller D., Mandal N., Diaz M., Fell J., Fini M.E., Alfonso E.C. (2009). Molecular identification of T4 and T5 genotypes in *Acanthamoeba* keratitis patients. J. Clin. Microbiol..

[70] Risler A., Coupat-Goutaland B., Pelandakis M. (2013). Genotyping and phylogenetic analysis of *Acanthamoeba* isolates associated with keratitis. Parasitol. Res..

[71] Booton G.C., Visvesvara G.S., Byers T.J., Kelly D.J., Fuerst P.A. (2005). Identification and distribution of *Acanthamoeba* species genotypes associated with nonkeratitis infections. J. Clin. Microbiol..

[72] Wang H., Masters S., Hong Y. (2012). Effect of disinfectant, water age, and pipe material on occurrence and persistence of Legionella, mycobacteria, *Pseudomonas aeruginosa*, and two amoebas. Environ. Sci. Technol..

[73] Niyyati M., Lasgerdi Z., Lorenzo-Morales J. (2015). Pathogenic free-living amoebae from water sources in Kish Island, Southern Iran. Microbiol. Insights.

[74] Behniafar H., Niyyati M., Lasjerdi Z. (2015). Molecular characterization of pathogenic *Acanthamoeba* isolated from drinking and recreational water in East Azerbaijan, Northwest Iran. Environ. Health Insights.

[75] Kao P.M., Hsu B.M., Chen N.H., Huang K.H., Huang S.W., King K.L., Chiu Y.C. (2012). Isolation and identification of *Acanthamoeba* species from thermal spring environments in southern Taiwan. Exp. Parasitol..

[76] De Jonckheere J.F. (2005). The isolation of *Naegleria italica* from Peru indicates that this potentially pathogenic species occurs worldwide. Parasitol. Int..

[77] Ithoi I., Ahmad A.F., Nissapatorn V., Lau Y.L., Mahmud R., Mak J.W. (2011). Detection of *Naegleria* species in environmental samples from Peninsular Malaysia. PLoS ONE.

[78] Rompre A., Servais P., Baudart J., de-Roubin M.-R., Laurent P. (2002). Detection and enumeration of coliforms in drinking water: Current methods and emerging approaches. J. Microbiol. Methods.

[79] Mahto B., Goel S. (2008). Bacterial survival and regrowth in drinking water systems. J. Environ. Sci. Eng..

[80] Ahmed I., Rafique T., Hasan S.K., Khan N., Khan M.H., Usmani T.H. (2012). Correlation of fluoride in drinking water with urine, blood plasma, and serum fluoride levels of people consuming high and low fluoride drinking water in Pakistan. Fluoride.

[81] Firempong C.K., Nsiah K., Awunyo-Vitor D., Dongsogo J. (2013). Soluble fluoride levels in drinking water—A major risk factor of dental fluorosis among children in Bongo community of Ghana. Ghana Med. J..

[82] Kocher D.K., Gupta A., Sahota P.P. (2014). Incidence of water borne protozoans and their correlation with faecal indicator bacteria in drinking water. Indian J. Appl. Res..

[83] Azman J., Init I., Wan-Yusoff W.S. (2009). Occurrence of *Giardia* and *Cryptosporidium* (oo)cysts in the river water of two recreational areas in Selangor, Malaysia. Trop. Biomed..

